# Evaluation of different treatment modalities on the efficacy of hydroxypropyl Guar (HP-Guar) formulation on tear film stability (TFS) in subjects exposed to adverse environmental conditions

**DOI:** 10.1186/s12886-023-02977-3

**Published:** 2023-05-22

**Authors:** Ali Abusharha, Ian E. Pearce, Tayyaba Afsar, Ali Alsaqr, Raied Fagehi, Suhail Razak

**Affiliations:** 1 Department of Optometry, College of Applied Medical Sciences, King Saud University, Riyadh, Saudi Arabia; 2 Vision Sciences, Glasgow Caledonian University, Glasgow, Scotland, UK; 3 Department of Community Health Sciences, College of Applied Medical Sciences, King Saud University, Riyadh, Saudi Arabia

**Keywords:** Dry eye, Tear film stability, Tear evaporation rate, Controlled environment chamber, Desiccating environment, Hydroxypropyl guar, Treatment modalities

## Abstract

The study aimed to assess the efficacy of hydroxypropyl guar (HP) formulation (Systane) to protect tear film parameters under desiccating environment using protection and relief treatment modalities. The subjects were exposed to adverse environmental conditions using a Controlled Environment Chamber (CEC) where the relative humidity (RH) was 5% and the ambient temperature was 21 °C and screened for Tear break-up time (TBUT), Tear film evaporation rate (TFER) and lipid layer thickness (LLT) using the HIRCAL grid, Servomed EP3 Evaporimeter and Keeler’s TearScope-Plus respectively. Significant improvement in LLT was noticed in the protection modality. The mean tear film evaporation rate doubled after exposure to the humidity of 5% to a value of 105.37 g/m^2^/h (0.29 µl/min). All subjects displayed a significant reduction in non-invasive tear break-up time (NITBUT) with a mean NITBUT of 7.7 s after exposure to a desiccating environment for 15 min. A significant increase in NITBUT after the instillation of the drops was recorded in both methods. The results obtained from this study showed that a solution containing HP-Guar significantly improves tear film parameters under a desiccating environment. Apart from the tear evaporation rate, all tear parameters showed improvement after the use of HP-Guar eye drops. It is evident that tear film parameters respond differently to the management modalities and using CEC has the potential to provide researchers with a readily available method to evaluate the efficiency of tear supplementation.

## Key points

### What is known

- Artificial tears which contain electrolytes such as sodium chloride have been widely used to manage tear film disorders.
- Thinning of the tear film lipid layer in an adverse environment could cause an alteration in tear stability and evaporation rate.
- Artificial tears and lubricants are commonly used to treat the signs and symptoms of dry eye. however, the main drawback of most of these formulations is the short retention time as the solution can easily drain via the lacrimal drainage system.
- Hydroxypropyl guar (HP-Guar) drops (Systane) can extend the tear film break-up time and enhance tear film stability in dry eye patients.
- The efficiency of HP-Guar in protecting the tear film parameters in a desiccating environment. Previous studies have used several techniques to create a dry environment and simulate dry conditions such as aeroplanes cabin or air-conditioned offices. However, some of these techniques have various limitations. One of the problems was the failure to fully monitor and control relative humidity around the eye

### What is new

The ability of Systane drops in desiccating conditions has been assessed previously by various techniques, however, there were many deficiencies in the treatment module such as inaccurate control of relative humidity (RH) and creating an additional airflow effect on the ocular surface.

A new technique of inducing signs and symptoms of dry eye by exposing normal subjects to the desiccating environment (5% RH) was utilized to assess the efficacy of a formulation containing HP-Guar.

We assess the ability of dry eye tear supplements to protect tear film parameters under low humidity conditions; and secondly, to discover the best method to use these supplements to either protect or relieve tear film disturbance which may occur after exposure to desiccating environmental conditions.

To produce different environmental conditions a controlled environment chamber was used.

The “protection” method is designed to assess HP-Guar's role in the protection of the tear film structure by using the product before the tear film is exposed to adverse environmental conditions. In this modality, the drop with a volume ranging between 40 and 45 µl was instilled first, and then the tear film was exposed to the adverse environmental conditions (5%RH/21 °C) for 15 min before the tear parameters were observed (Fig. 2-A)

The “relief” mechanism is designed to assess the ability of dry eye drops to relieve the symptoms of the dry eye once induced. In the relief method, HP-Guar (Systane) significantly improves tear film parameters under a desiccating environment. Apart from the tear evaporation rate, all tear parameters showed improvement after the use of HP-Guar eye drops. Tear film parameters respond differently to the management modalities that were used in this study (protection and relief)

Controlled Environment Chamber (CEC) has the potential to provide researchers with a readily available method to evaluate rapidly the efficiency of tear supplementation. By using the CEC, tear film parameters that are typical to those with dry eye patients could be simulated easily in the laboratory environment. This new method allows further evaluation of tear film parameters and dry eye treatment protocols in labs before trying it on dry eye patients.

## Introduction

There are many treatments used for the management of Dry Eye Disease (DED) [1, 2]. Artificial tears and lubricants are commonly used to treat the signs and symptoms of dry eye [3]. Artificial tears which contain electrolytes such as sodium chloride have been widely used to manage tear film disorders [4]. Oil–water emulsion eye drops have been found to help to manage evaporative dry eye and enhance tear film stability [5]. The main drawback of most of these formulations is the short retention time as the solution can easily drain via the lacrimal drainage system [6]. Attempts have been made to increase the contact time between tear film substitutes by increasing the viscosity of the dry eye solution. However, this kind of formulation is more likely to cause vision disturbance, making it difficult to use especially in the daytime. Also, it is uncomfortable as they transfer the sheer force of the blink to the ocular surface [7]. The reduction of lipid layer thickness at low RH has also been reported [8, 9]. Korb et al. demonstrated that the lipid layer thickness (LLT) of tear film increased significantly in dry eye patients when the humidity increased [10]. Thinning of the tear film lipid layer in an adverse environment could cause an alteration in tear stability and evaporation rate. The tear film is a thin complex structure composed of three separate layers: mucus, water and oil. Tear film plays a vital role in maintaining a healthy ocular surface and helps produce a clear retinal image. Numerous tests have been developed to examine tear dynamics and diagnose tear film abnormalities [11].

Hydroxypropyl-Guar (HP-Guar) gel-able lubricant eye drops are a gelling polymer formulation that has recently been used to enhance solution retention. Systane (Alcon Laboratories, Fort Worth, Texas, USA) is a commercial tear supplement containing HP-Guar. Systane also contains polyethene glycol 400 (0.4%) and propylene glycol (0.13%). The ability of Systane to extend the tear film break-up time and enhance tear film stability is well documented [12–14]. Systane use significantly reduced corneal staining after 28 days of treatment [15]. In another study, the lubricity of five over-the-counter eye drops was assessed using a reciprocating friction device. It was found that Systane reduced the coefficient of friction significantly compared to other eye drops [16, 17]. Further studies comparing different dry eye drops showed a significant improvement in corneal and conjunctival staining, a decrease in ocular symptoms and ocular discomfort among dry eye patients after Systane use [18]. It is believed that a formulation containing HP-Guar presents an additional benefit for tear film in dry eye patients [18, 19]. The efficacy of Systane in desiccating conditions has also been assessed previously by various techniques, however, there were many ineffectualities in the treatment module such as inaccurate control of RH and creating an additional airflow effect on the ocular surface. Hence, further investigations are needed to fully assess the efficiency of HP-Guar in protecting the tear film parameters in a desiccating environment. Previous studies have used several techniques to create a dry environment and simulate dry conditions such as aeroplanes cabin or air-conditioned offices. However, some of these techniques have various limitations. One of the problems was failure to fully monitor and control relative humidity around the eye. Therefore, these techniques assess the tear film under a range of RH condition rather than at a fixed RH. Also, the lack of an ability to expose the eye to dry conditions for a long period of time to evaluate the impact of extended exposure to desiccating environment on the tear film. In the current study, a new technique of inducing signs and symptoms of dry eye by exposing normal subjects to desiccating environment (5% RH) was utilized to assess the efficacy of a formulation containing HP-Guar. The aim of this study was twofold. Firstly, to assess the ability of dry eye tear supplements to protect tear film parameters under low humidity conditions; and secondly, to discover the best phase to use these supplements to either protect or relieve tear film disturbance, which may occur after exposure to desiccating environmental conditions.

## Materials and methods

### Eye drops

In the current study, SYSTANE® Lubricant Eye Drops (Alcon Laboratories, Fort Worth, Texas, USA)– Original- was used. We have selected this product intending to evaluate the ability of hydroxypropyl Guar (HP-Guar) to protect tear film parameters against adverse environments. We selected this formulation because Other Systane products (such as SYSTANE® Balance) contain mineral oils which may interfere with HP-Guar.

### Controlled environment chamber (CEC)

To produce different environmental conditions a controlled environment chamber was used – an isolated room 3 × 3x2 metres designed and constructed by Weiss-Gallenkmap, (Loughborough, UK). The CEC can produce any temperature between 5 and 35ºC (± 2 ºC) and relative humidity (RH) between 5 and 95% (± 3%). The chamber is fitted with a Rotronic direct reading combined temperature /relative humidity sensor for determination and control of humidity and temperature. The temperature and humidity can be fully controlled by the operator using the control panel of the chamber. An accurate temperature can be achieved using the air conditioning unit (ACU). The ACU is sited with refrigeration and electrical heating units that allow continuous control of the ambient temperature in the room. To control the RH inside the room, a vapour phase generator (VPG) and desiccant dehumidifier are integrated and situated at the back of the chamber. The RH is increased by producing water vapour in the VPG humidifier which is then introduced into the CEC; while the dehumidifier which is equipped with a Lithium Chloride desiccant rotor helps to absorb moisture from the air and reduce the RH. The RH is controlled automatically by the chamber humidity controller by switching on or off the humidification system and modulating the power to the dehumidification unit.

### Study design

This non-randomized, observational, and comparative study enrolled a total of 12 healthy normal subjects (3 females, 9 males, 29 ± 4 years). All human procedures were performed following the ethical standards of the committee responsible for human experimentation (Institutional and National), and with the Helsinki Declaration of 1975, as revised in 2019 [20]. Approval for the study was obtained from the Institutional Review Board, Glasgow Caledonian University Ethics Committee, (GCU1321/34/A). Written and oral informed consent including an explanation of the study procedures and requirements were signed by all participants.

**Fig. 1:**
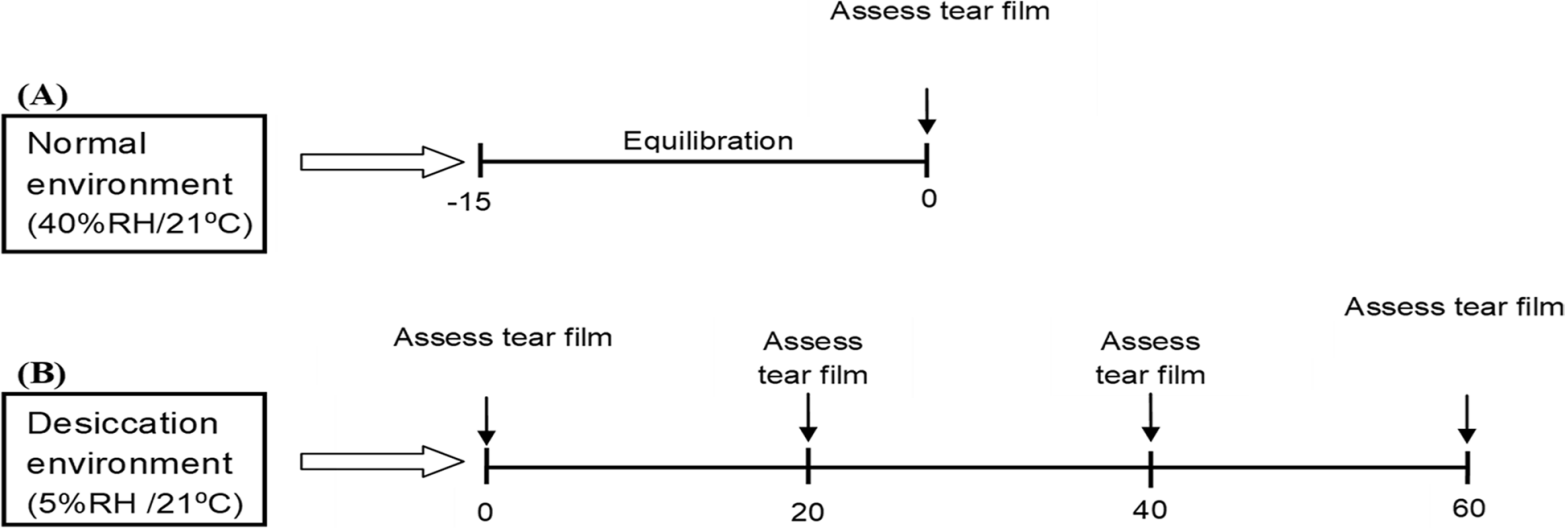
Study design. **A** observation of the subject in a normal environment (40% RH/21ºC), Tear parameters included lipid layer thickness (LLT), evaporation rate and tear breakup time were assessed after 15 min of room adaptation. **B** At low humidity conditions (5% RH/21ºC) tear film physiological investigations were carried out immediately (0 min) and then at 20, 40 and 60 min time points. The tear parameters described above were assessed at all time points

All subjects were invited for screening visits where tear production and tear break-up time was assessed. The inclusion criteria for the subjects were a NITBUT value of more than 10 s. If the subjects met the inclusion criteria the basal measurements of tear film parameters at normal conditions (40%RH/21 °C) were carried out during the screening visit. The instillation of eye drops could result in an immediate increase in tear volume. This could be varying between subjects depending on the rate of tear turnover including the secretion and drainage of the tears. In addition to that residual tears in the lower conjunctiva also differs from subject to subject and could affect that. In the current study, all measurements were performed five minutes following the instillation of eye drops to standardize the tear film volume status. Subjects with a history of ocular surgery, ocular disease or who wear contact lenses were excluded from this study. Figure 1 summarized the study design.

### Treatment modalities

For control and monitoring of the relative humidity and temperature, all investigations were carried out in a Controlled Environment Chamber (CEC). The subjects were exposed to adverse environmental conditions where the RH was 5% and the ambient temperature was 21 °C.

**Fig. 2:**
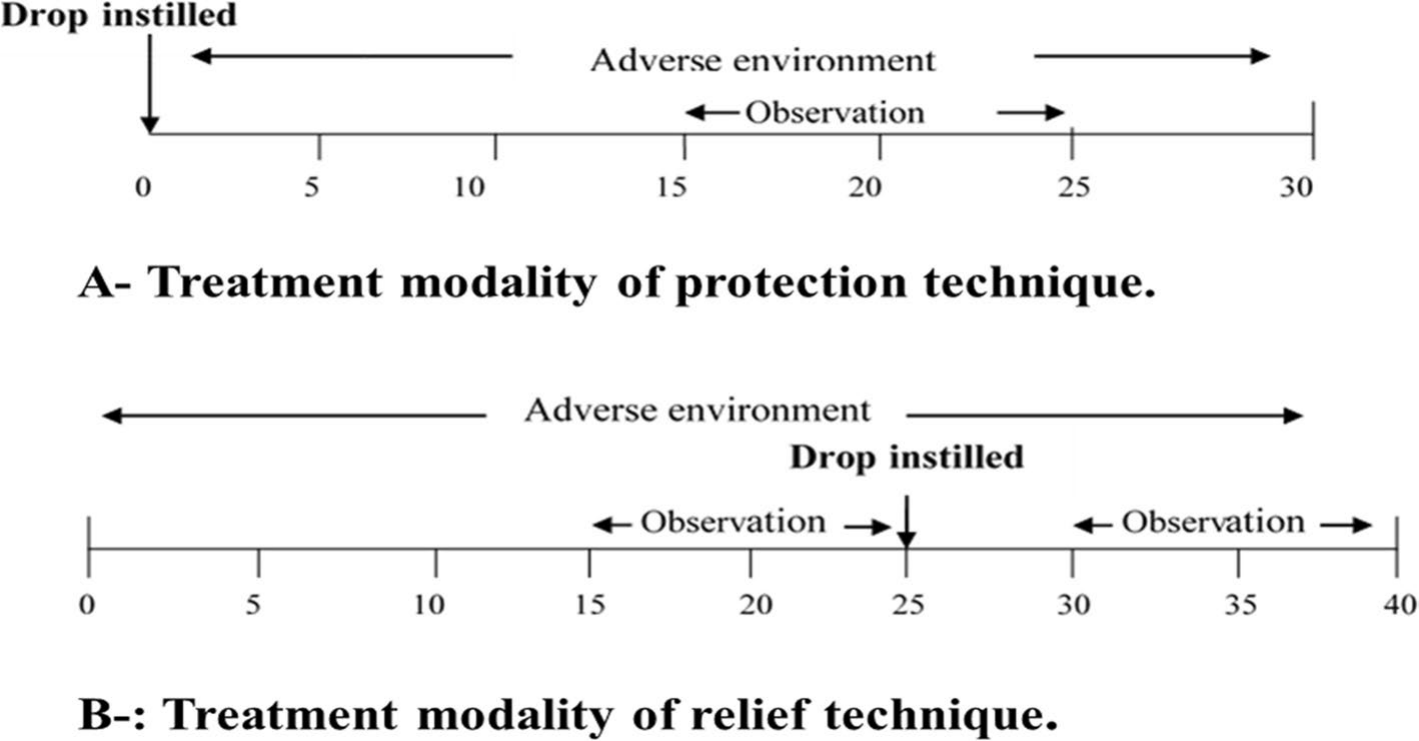
**A**-Treatment modality of the protection technique. The drop was instilled before tear film exposure to a dry environment (5%RH/21 °C). The tear film parameters were assessed after 15 min of exposure. **B**- Treatment modality of relief technique. The subject was exposed to the adverse environment (5% RH/21 °C) for 15 min, and the tear parameters were measured. The drop is instilled and then the tear parameters were measured again to see if any relief is experienced

Two different mechanisms were studied to evaluate the effect of treatment, protection and relief. The “protection” method is designed to assess HP-Guar's role in the protection of the tear film structure by using the product before the tear film is exposed to adverse environmental conditions. In this modality, the drop with a volume ranging between 40 and 45 µl was instilled first, and then the tear film was exposed to the adverse environmental conditions (5%RH/21 °C) for 15 min before the tear parameters were observed (Fig. 2-A).

The “relief” mechanism is designed to assess the ability of dry eye drops to relieve the symptoms of the dry eye once induced. In the relief method (Fig. 2-B) after the subject was exposed to the adverse environment (5%RH/21 °C) for 15 min, the tear parameters were measured. The drop was then instilled and then the tear parameters were measured again to see if any relief was experienced.

In addition to the screening visit, two visits were required to carry out all the investigations of the tear parameters for the right eye during the two modalities (protection and relief). The subjects were divided into two groups (A and B). Group A was seen in protection and Group B was seen in relief visit. Each group was then crossed over so that Group A was tested in the relief method and Group B at protection at their next visit. To monitor tear film behaviour during exposure to a dry environment, tear parameters were observed at 20 min intervals for one hour starting from the baseline assessment at 0 min (0 min, 20 min, 40 and 60 min). Control was to observe the same subjects at normal RH following 15 min equilibration.

### Parameter assessed

The tear evaporation rate was evaluated using a Servo-Med Evapometer [21]. Tear break-up time (NITBUT) and lipid layer thickness (LLT) were assessed using a Keeler TearScope-Plus. A Guillon and Guillon grading system was used to estimate the tear film LLT [22]. The brief detail of each procedure is described below.

### Non-invasive tear break-up time (NITBUT)

Tear film break-up time can be observed non-invasively by the HIRCAL grid (Bausch and Lomb Keratometer, UK) [23] and Keeler Tearscope Plus (Windsor, UK) [22]. The HIRCAL grid is a modified Bausch and Lomb Keratometer with the mire replaced by a white-on-black grid. The subjects were instructed to blink normally. The reflected image of the fine grid from the tear film was observed at the appearance of the first point of local disturbance or blurring of the grid lines. The time between the blink and the occurrence of disruption was recorded (in seconds) as tear break-up time. A smooth reflection from the tear film indicates a stable and regular tear film. When the tear film becomes thin and starts to break down the reflected image appears to be hazy and distorted. a NITBUT of less than 10 s is considered an unusual tear film (moderate dry eye – grade 2), while a NITBUT reading of fewer than 5 s is indicative of a severe dry eye (grade 3) [24]. The NITBUT value of 10 s provides a sensitivity of 82% and specificity of 86% for the diagnosis of DE [25]. Three measurements were recorded and the mean of three readings was calculated. In addition to the HIRCAL grid, a Keeler Tearscope Plus [22] was also used to assess tear film stability non-invasively during the main studies to save more space in the CEC with the advantage of allowing for observing tear film stability and lipid layer thickness. The Keeler Tearscope Plus is based on the principle of projecting and observing the reflected image of the grid from the tear film. The tearscope with fine grid insert was mounted in a slit-lamp biomicroscope to provide extra magnification of the reflected image. After the image was focused on the tear film, the subject was asked to blink normally while the image of the grid was observed. The first point of disruption was noted. The time between the blink and when the image first began to distort was recorded three times and the mean value was calculated.

### Tear film evaporation rate (TFER)

**Fig. 3:**
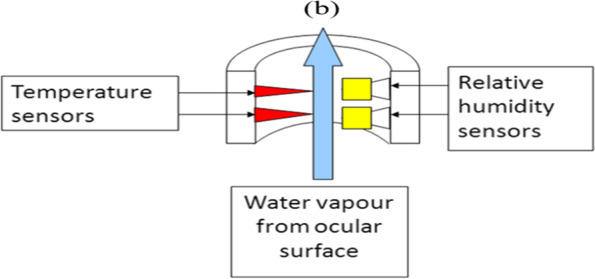
A schematic diagram shows the temperature and humidity sensors fitted into a probe to calculate the water vapour pressure. The evaporation rate can then be calculated

Evaporation of tear film was measured using a modified Servomed EP3 Evaporimeter (Servo Med, Varberg, Sweden) [21]. A pair of sensors, one for humidity and one for temperature are fitted into a probe mounted on a slit lamp. The probe was attached to a modified swimming goggle at a known distance from the eye to isolate the ocular surface from air currents and the ambient environment and to avoid direct contact with the ocular surface. The subject was instructed to hold the goggle securely on their right eye to ensure no gap between the skin surface and the goggle. Measurements were taken twice for each subject, one with an open eye and the other with a closed eye. Measurements with an open eye represent the evaporation of both tear film and skin within the goggle, while the closed eye shows the evaporation from the skin only. The subjects were asked to blink normally and look at a fixation point while the test was carried out for the open eye. The schematic diagram (Fig. 3) shows the temperature and humidity sensors fitted into a probe.

### Measurement of lipid layer thickness (LLT)

**Fig. 4:**
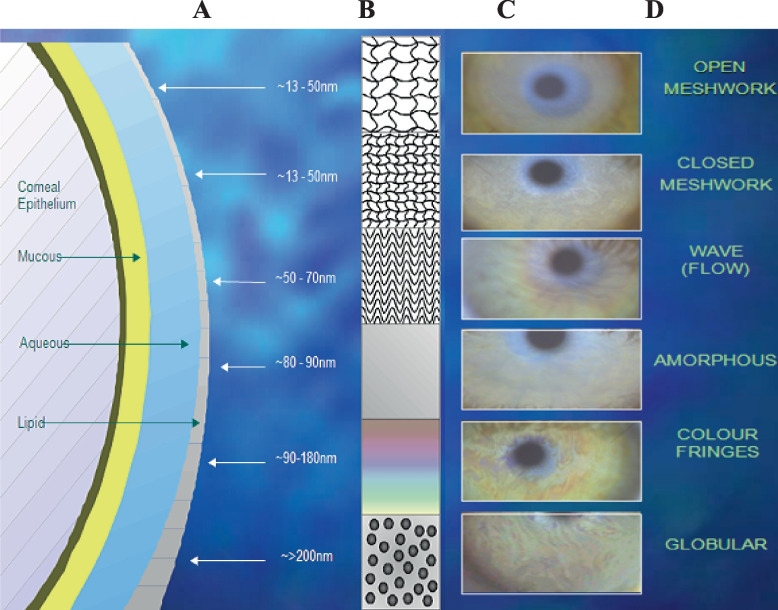
Tear film grading images show the thickness in nanometres for each pattern based on the grading system of Guillon and Guillon. **A** Schematic of tear film layers and the thickness of lipid layers in nm. **B** Schematic of the lipid layer appearance with different thicknesses. **C** Photos of lipid layer fringes at different patterns. **D** Names of fringes pattern according to the lipid layer thickness

In this study, a Tearscope Plus (Keeler Ltd, Berkshire, UK) in combination with a non-illuminated biomicroscope was used to observe the quality and thickness of the tear film lipid layer [22]. The Tearscope Plus is a wide-angle lighting system with a cold-cathode light source. The subjects were seated at a slit lamp and asked to blink normally and fixate on the light source straight ahead. The system was then focused carefully to obtain a sharp and clear image of the interferometric patterns. The grading system of Guillon and Guillon was used to estimate the classification and therefore the thickness of the lipid layer (Fig. 4) [22].

### Statistics

All data were statically analysed using PASW Statistics version 18 (IBM corporation, Somers, NY, USA). A test of normality was carried out first using a Kolmogorov–Smirnov test. Normally distributed data were compared using a repeated measured ANOVA and Tukey’s posthoc test. Data not normally distributed were compared using Friedman’s test and a post-hoc Wilcoxon rank sum test. Correlation between parameters was derived using Pearson’s correlation for normally distributed data, while data not following normal distribution were correlated using Spearman’s Rho test.

## Results

Twelve healthy subjects were enrolled in this study. A summary of the data (mean and standard deviation) of the values of tear film parameters measured at 40 and 5%, protection and relief are shown in Table 1.

**Table 1:** Mean and standard deviation of the tear parameters measured at 5% RH, and 40% RH, and after instillation of the Systane eye (HP-Guar) drop either in protection or relief mode

	**EVAP (g/m**^**2**^**/h)**	**NITBUT (sec)**
40% RH	52.98 ± 9.15	15.0 ± 2.0
5% RH	105.4 ± 37.32	8.0 ± 3.00
Protection	96.91 ± 41.45	10.0 ± 2.00
Relief	99.15 ± 28.40	10.0 ± 2.00

*Evap* Evaporation rate, *NITBUT* Non-invasive tear break-up time, *RH* Relative humidity

### Tear film evaporation rate

**Fig. 5:**
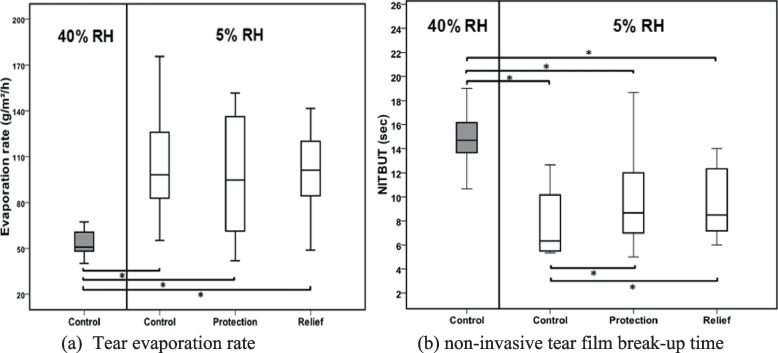
**a** Tear evaporation at 40 and 5% relative humidity and post-instillation of HP-Guar at different protocols (relief and protection). The evaporation rate was significantly higher at 5% RH even after the instillation of eye drops in all visits (*n* = 12). The box represents the interquartile range that contains 50% of the values. The whiskers are lines that extend from the box to the highest and lowest values, excluding outliers (O) that are 1.5 to 3 box lengths from the upper and lower edge of the box. The line across the box indicates the median value. Pairwise significant differences are indicated by (*). **b** A box plot showing non-invasive tear film break-up time at normal and dry environmental conditions (*n* = 12). NITBUT was significantly shorter under dry conditions. Significant increase in mean NITBUT was seen after the instillation of HP-Guar in both visits. Box-Plot details as described in Figs. 5 and 6. Pairwise significant differences are indicated by (*)

The mean tear film evaporation rate in a normal environment (40%/21ºC) was 52 ± 9.15 g/m^2^/h. There was a significant increase in the evaporation rate when the tear film was exposed to the dry conditions (*p* = 0.003) (Fig. 5 a) Tear film evaporation doubled under the dry conditions to a rate of 105.37 ± 37.32 g/m^2^/h. There was a reduction in tear evaporation rate with the installation of HP-Guar in comparison with 5%. However, this reduction was not statistically significant in both the protection (96.92 ± 41.45 g/m^2^/h) (*p* = 0.91) and relief (99.51 ± 28.40 g/m^2^/h) (*p* = 0.72) method for HP-Guar.

### Non-invasive tear breakup time (NITBUT)

From the data in Fig. 5b, it is apparent that tear film stability (TFS) was significantly affected by the adverse environment. Exposure to the dry condition resulted in a reduction of NITBUT (8 ± 3 s) compared with the normal conditions (15 ± 2 s). Pairwise analysis showed that NITBUT improved significantly after instilling HP-Guar. Both methods succeeded in enhancing tear stability under a desiccating environment. Mean NITBUT increased to 10 s in the protection and relief methods, respectively. However, no significant difference in NITBUT was observed between the two methods (*p* = 0.58) of using HP-Guar.

### Lipid layer thickness (LLT)

**Fig. 6:**
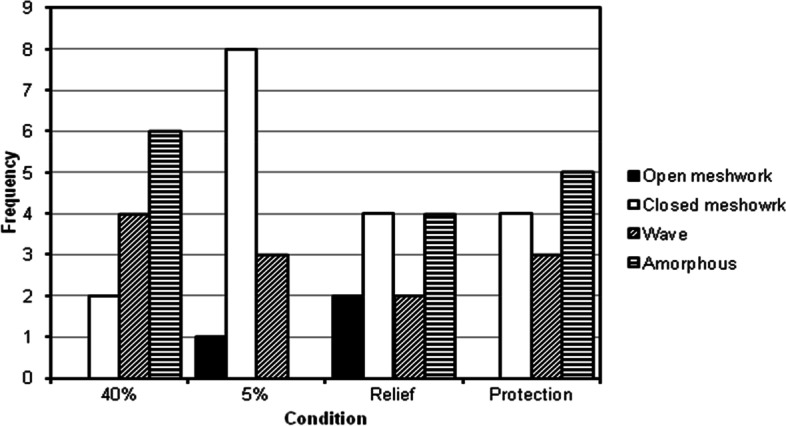
Distribution of lipid layer patterns observed under each environmental condition

Figure 6 shows the distribution of lipid layer patterns observed under each environmental condition and following the use of HP-Guar for relief and protection. The lipid layer thickness was adversely affected by the dry conditions. A significant reduction in LLT was observed (*p* = 0.04) at 5%RH compared with 40% RH*.* An increase in LLT was seen following the instillation of the drop in the protection technique (*p* = 0.018). No significant difference in LLT was found after the instillation of the eye drop in the relief method (*p* = 0.12).

## Discussion

A considerable number of studies have reported that not only outdoor working places but also indoor or closed buildings can expose workers to an adverse dry environment [26, 27]. Unfortunately, these environmental factors are unavoidable. Recent evidence suggests that a relationship exists between increasing detrimental health effects and time spent in an artificially ventilated workplace [28]. An ultra-dry environment has been recorded on long flights and in some workplaces such as high-tech devices factories, where the RH is less than 5% [29]. Eye irritation, upper airways symptoms, headache, dry throat and itchy skin have been commonly reported among employees working in such buildings [28, 30]. This study investigated the effect of a tear film supplement to treat tear film changes during exposure to an adverse dry environment. New techniques were tested to assess the efficiency of HP-Guar to either protect or relieve tear film parameters during exposure to a desiccating environment. Also, the effect of the desiccating environment on the human tear film was assessed.

Low humidity indoor environment was simulated using a Controlled Environmental Chamber at the Tear Physiology unit, Glasgow Caledonian University. The tear film was exposed to low humidity and the changes in tear parameters were monitored. Then, attempts were made to find out the optimal method to recover normal tear behaviour during exposure to a dry environment. The dry eye tear substitute was used in different ways to ascertain whether tear supplements can save the tear film against such an adverse environment. Two different methods were applied, where the drops were instilled before exposure in the “protection” method and post-exposure in the “relief” method.

A thinner lipid layer thickness (LLT) was observed in this study when the tear film was exposed to a desiccating environment. Changes in the tear film lipid layer may have resulted from an alteration in the tear protein and the lipid polar phase as a result of the change in the RH [31, 32]. We noticed a significant improvement in the lipid layer pattern when the eye drop was instilled before exposure to the dry environment (protection). Other studies observing the LLT using a lipid layer interferometer have suggested an increase of 16% in the LLT following the instillation of Systane eye drops [33]. The average thickness of the lipid layer increased from 61.5 to 71.3 nm after the installation of the HP-Guar agent at a humidity range between 35 and 50% at an ambient temperature of 25ºC [33]. A layer of soft gel is formed when HP-Guar is applied to the eye as a result of the change in pH when it encounters the tear film. The gel layer spreads over the ocular surface, enhancing the spread of the tear film [34]. The increase in LLT is unlikely induced by an increase in lipid secretion but could result from the improvement of lipid spreading and/or an increase in the thickness of the underlying aqueous layer [33, 35].

According to the Definition and Classification Subcommittee of the international workshop on the dry eye (DEWS), evaporative dry eye is considered one of the main types of dry eye causes [24].

In the current study, one of the parameters monitored was the tear evaporation rate. The mean tear evaporation rate at normal environment conditions was 52.98 g/m^2^/h (0.15 µl/min). This is in agreement with the suggested weighted mean of normal evaporation rate (0.14 ± 0.07 µl/min) reported over the past 30 years, stated by Tomlinson et al. [36]. This value doubled after exposure to the humidity of 5% to a value of 105.37 g/m^2^/h (0.29 µl/min). It should be considered that this value is higher than the suggested cut-off value of the evaporation rate between normal and dry eye patients (0.20 to 0.22 µl/min) [36]. Excessive evaporation rate in a dry environment has been previously reported [37, 38]. The evaporation rate was found to double when the RH decreased from 40 to 20% [38]. Another study showed that a 10% decrease in RH (from 35–45 to 25–35%) resulted in an average evaporation rate increase of 30 and 33% in normal and dry eye patients, respectively [39]. However, elevation in the evaporation rate was expected as a thin lipid layer was also observed in this study after exposure to low humidity. The evaporation rate remained high in dry conditions (5% RH) even after the installation of HP-Guar eye drops. Previous work by Uchiyama et al. showed that the instillation of Hydroxypropyl Guar (Systane) resulted in a minimal reduction in evaporation rate among dry eye patients at RH of 25–35%, where the evaporation rate dropped from 0.15 to 0.12 µl/min [40]. However, another investigation by the same group showed contradictory results with no significant change in tear film evaporation pre and post-instillation of Systane under different RH conditions in dry eye patients [41]. The disparity in the result might be due to several limitations including inaccurate control of RH and exposure of the eye directly to a dry airflow that may induce an extra effect on the evaporation rate.

Throughout this study, we noticed that tear film stability was adversely affected by the dry environment. All subjects displayed a significant reduction in non-invasive tear break-up time (NITBUT) with a mean NITBUT of 7.7 s after exposure to a desiccating environment for 15 min. It should be noted that the suggested mean of inter blink interval is 7.5 s [42]. The potential for ocular surface desiccating and damage increases when tear break-up time is shorter than the blink interval. The study demonstrates that there is a significant increase in NITBUT after the instillation of the drops in both methods.

In the current study, we found that the instillation of Systne increased NITBUT from 8 to 10 s under 5% RH. So far there have been no studies which assess the efficiency of HP-Guar to enhance tear stability under adverse dry environments. However, many reports have shown a significant improvement in tear film stability either immediately or after prolonged use of HP-Guar eye drops among dry eye patients [43–45]. Previous reports have found that HP-Guar provides immediate longer fluorescein Tear break-up time (TBUT) in dry eye patients where the mean TBUT increases from 10 to 19.50 s after instilling of HP-Guar [45]. Earlier reports have shown that TBUT was prolonged after 28 days of Systane use among dry eye patients. Mean TBUT was significantly improved from 6.9 s at the baseline visit to 8.5 after using Systane for 28 days [44]. The improvement in tear film stability could be due to the formation of a soft gel-like shield after the instillation of HP-Guar due to a change in pH. The formation of a structured polymer network on the ocular surface enhances the stability and spread of the tear film over the ocular surface [46].

## Conclusion

The overall results of this study suggest that tear film parameters were adversely influenced by exposure to a dry environment. The results obtained from this study showed that a solution containing HP-Guar (Systane) significantly improves tear film parameters under a desiccating environment. Apart from the tear evaporation rate, all tear parameters showed improvement after the use of HP-Guar eye drops. Tear film parameters respond differently to the management modalities that were used in this study (protection and relief). In the protection method, tear film osmolarity and lipid layer thickness were protected against a dry environment, while in the relief mode, an improvement in tear production and a decrease in ocular surface temperature were seen. This suggests that both mechanisms could be effective for normal subjects but each method showed a different effect on certain tear parameters. With consideration of the major group of dry eye (aqueous deficiency and evaporative dry eye), HP-Guar performance could be maximised for the management of exposure to adverse environments by using a treatment protocol that targets the most affected parameters in each group of dry eye patients. However, as the tear film in dry eye subjects may behave differently, further investigation on dry eye patients with consideration of the dry eye subtype will be needed to confirm this finding.

The present study demonstrates that using CEC has the potential to provide researchers with a readily available method to evaluate rapidly the efficiency of tear supplementation. By using the CEC, tear film parameters that are typical to those with dry eye patients could be simulated easily in the laboratory environment. This new method allows further evaluation of tear film parameters and dry eye treatment protocols in labs before trying it on dry eye patients.

However, tear evaporation, which is one of the most critical tear film parameters, was not protected in these adverse conditions. In addition, although some tear parameters improved following HP-Guar use, tear stability and evaporation rate recorded values that would be considered out of the normal range. Therefore, further investigation using other solutions with a formula that targets the lipid layer and evaporation rate of tear film may be needed to control the evaporation rate under such desiccating conditions.

## Data Availability

All the relevant data has been provided in the manuscript. Supplementary datasets used and/or analyzed during the current study are available from the corresponding author upon reasonable request.
